# Considering research waste in economic evaluations of low-dose computed tomography screening for lung cancer

**DOI:** 10.1017/S0266462326103687

**Published:** 2026-03-30

**Authors:** Jaime L. Peters, Tristan Snowsill, Sophie Robinson, Edward Griffin, Christopher Hyde

**Affiliations:** 1 Exeter Test Group, Department of Health and Community Sciences, University of Exeter, UK; 2 Health Economics Group, Department of Health and Community Sciences, University of Exeter, UK; 3 PenTAG, Department of Public Health and Sport Sciences, University of Exeter, UK; 4 Associate of the Exeter Test Group, Department of Health and Community Sciences, University of Exeter, UK

**Keywords:** lung neoplasms, early detection of cancer, cost–benefit analysis, access to information, Tomography, X-Ray Computed

## Abstract

With the ongoing discussion around the implementation of low-dose computed tomography (LDCT) screening for lung cancer, increasing numbers of model-based economic evaluations have been conducted. We systematically reviewed the published literature for economic evaluations of LDCT screening for lung cancer in people at high risk, updating this review in 2024. We identified a total of 57 economic evaluations; two-thirds of which developed *de novo* decision-analytic models. In our most recent update, of the 22 economic evaluations identified, only one used a model published before 2021. There may be advantages to developing new models, but the huge possibility of research waste cannot be ignored. Better quality of reporting (both in terms of clarity and completeness), improving the availability of existing models, and comparative analysis of different models can help to advance modeling methods in areas of complexity, such as screening for cancer. Ultimately, this should lead to policy decisions based on the best available evidence.

## Introduction

Lung cancer often presents in later stages, when curative treatment is less likely. Low-dose computed tomography (LDCT) screening for lung cancer has been shown to be effective at reducing mortality in people at high risk (1). The extent to which LDCT screening for lung cancer is cost-effective has therefore received much attention.

In 2017, we systematically reviewed the published literature for cost-effectiveness studies of LDCT screening for lung cancer in high-risk populations (2). Since then, we have twice updated the review, running database searches in 2020 (3) and 2024 (now available in a report to funders, see Web Report at https://fundingawards.nihr.ac.uk/award/14/151/07). In total, we have identified 57 economic evaluations, set in 20 different countries. The earliest of those evaluations were published in 2001 and set in the United States (4;5), with more recent ones set in Asia (6;7), particularly China (8;9). As the evidence and discussion on the implementation of LDCT screening for lung cancer in high-risk populations has evolved, it is not surprising to observe so many evaluations in different territories. Many of these published economic evaluations may be used to support national decision making on LDCT screening for lung cancer, with a number of articles being explicit about this aim (e.g., in Hungary (10), the Netherlands (11;12), Korea (6), Taiwan (7), and Austria (13)). According to the Lung Cancer Policy Network (14), nine countries have national LDCT screening program for lung cancer.

In our 2020 update, we highlighted variation across the 35 published model-based economic evaluations, noting 13 studies that had either used an existing model in their evaluations or taken very similar approaches (e.g., Whynes (15), Field et al. (16), and Hinde et al. (17) for the UK setting), see Table 1. When we updated the review in 2024 with an additional 22 economic evaluations, we were surprised to find that only one of these newer evaluations (29) had used a model published before 2021 (26). However, three of the newer models had been used in more than one economic evaluation, see Table 1.

**Table 1.** Economic evaluations where existing models were used

Originator	Publications using model
Whynes (15)	Field et al. (16) Hinde et al. (17)
Marshall et al. (4)	Marshall et al. (5)
Pyenson et al. (18)	Pyenson et al. (19) Villanti et al. (20)
Lung Cancer Outcomes Simulator model (21)	Criss et al. (22) Toumazis et al. (23)
Lung Cancer Policy Model (24)	McMahon et al. (25) Criss et al. (22)
MISCAN-Lung model (26)	ten Haaf et al. (27) Tomanaga et al. (28) Criss et al. (22) Tomonaga et al. (29)
University of Michigan Lung Cancer Screening model (30)	Criss et al. (22)
Pan et al. (31)	ten Berge et al. (13) ten Berge et al. (32) ten Berge et al. (12)
Yuan et al. (33)	Yuan et al. (34)
Zhao et al. (35)	Zhao et al. (9)

Overall, we estimated that across the 57 total number of economic evaluations, there were 38 *de novo* models. These models, as far as we could determine, had not previously been used to evaluate the cost-effectiveness of lung cancer screening. Given that there had been over 20 years of published research evaluating the cost-effectiveness of LDCT screening for lung cancer, this was unexpectedly high. A similar observation has been made in the evaluation of screening for colorectal cancer: Adair et al. (36) identified 52 de novo models in a systematic review of 78 model-based economic evaluations.

## Avoiding research waste

At the outset, developing a *de novo* model, rather than adapting an existing model, may appear to have a number of advantages: It can be developed to answer the specific question of interest for the relevant policy maker, and there are no issues in trying to decide which, of a number of existing models, would be the most appropriate one to use. Furthermore, no model is provably correct, and standard peer review of a model does not necessarily validate it. If important aspects of the decision problem have not been captured in models that are repeatedly used, this is likely to lead to biased cost-effectiveness estimates, resulting in potentially misleading conclusions on which policy decisions are made. Initially, it may appear that a simple model is sufficient and can be developed quickly and cheaply (assuming there is some in-house expertise); for example, there are no costs for access to an existing model and no time spent trying to understand someone else’s model (which may have been developed in unfamiliar software).

However, we argue that models to evaluate cancer screening programs are necessarily complex. A model that has been developed quickly and cheaply is unlikely to have addressed the many important aspects of cancer screening. We require cancer screening models to appropriately consider features such as overdiagnosis, lead-time bias, length bias, and incidental findings, as well as being able to consider variation in how screening is delivered (e.g. targeting, stratification, and timing). Only models containing natural history components can achieve this. The development and validation of these models is a significant undertaking, often requiring years of sustained investment, as demonstrated by the Cancer Intervention and Surveillance Modeling Network (CISNET; https://cisnet.cancer.gov/).

CISNET brings together researchers funded by the National Cancer Institute in the United States who are using modeling to evaluate interventions for the prevention, screening, and treatment of different cancers. CISNET takes a comparative approach to modeling, aiming to use multiple models to address the same research question (22;37). This approach has the potential to be more informative than relying on a single model: When models provide similar results, this offers reassurance, but when results are not similar, this can help to identify important differences in the structure or implementation of the models.

In our review, very few publications commented on the availability of the models [one as open access (2), one as being proprietary (38), and two having code available upon (reasonable) request (11;39)]. Some earlier papers do report in detail many of their equations, for example, Whynes (15) and Black et al. (40), while Goffin et al. (41;42) provide a website for the OncoSim model, but the link is no longer live.

Although there are often practical barriers to using an existing model, a minimum expectation in the development of a new model would be an evaluation of the strengths and limitations of existing models. Developing new models that rectify the limitations of previous models would present advancement in modeling methods and reduce the huge potential for research waste. Not only is there waste if researchers are “re-inventing the wheel” but also if the lessons from implementing more, necessarily, complex models are not taken into account. In our last update in 2024, there appeared to be limited evolution of the previously published and reviewed models. This is concerning given that we deemed many of the more recent *de novo* lung cancer screening models to be less sophisticated than older models. For instance, the proportion of *de novo* models that are Markov models has increased: 87 percent of the *de novo* models published since 2021 were Markov models, compared to 30 percent published before 2021. We found that Markov models are less likely to allow for the impact of overdiagnosis and lead time bias and in more recent publications we noted greater uncertainty as to whether all important costs and outcomes are identified. More generally, simpler models (*n* = 42) only evaluated one frequency of screening, while the more complex models evaluated a range of policy questions on frequency and duration of screening. Not considering multiple frequencies and durations of screening strategies could lead to inadequate policy decisions being made (43).

To sum up: Poor reporting and inaccessible models force policy makers to commission new models; however, with limited timescales, such models may not be fit for purpose, potentially leading to significant waste of public money if suboptimal policy decisions are subsequently made.

## The way forward

Future reports of model-based economic evaluations in cancer screening should meet the highest standards of transparency, and their underlying decision models should ideally be made open source or open access. Authors of already-published research should also consider placing models into the public domain.

Improving the completeness of reporting of models would allow better appraisal of the different models. In our review of evaluations for lung cancer screening, there were some studies where it was unclear whether they had used an existing model or developed a new model. For many studies, not all of the model information we sought could be extracted from the articles. The CHEERS statement was published in 2013 (updated in 2022) to help improve the quality of reporting of economic evaluations (44;45). Of the 45 evaluations in our review that were published from 2014 onwards, only six (13 percent) declared using the CHEERS statement to report their evaluation (2;8;9;29;46;47). However, by virtue of intending to be applicable to all methodologies for economic evaluation and not just decision modeling, the CHEERS statement does not require enough detail to make models reproducible. In the CHEERS 2022 explanation and elaboration (48), it is recommended that authors of reports of modeling studies may wish to follow separate reporting guidelines (49–51). As many journals have the capacity for publishing supplementary materials or appendices, we believe these opportunities should be more widely used to provide more detailed descriptions of models, the assumptions made, and the data used. However, it is very rare that any attempt at reporting is more transparent than simply making the model open source.

Making models available to other researchers, preferably being open source would help to improve future modeling studies. The current move toward making research data open and available is reflected in requirements from certain funders (e.g., United Kingdom Research and Innovation). Many publishers and journals have introduced open data statements (e.g., Springer Nature, BMJ Journals, IJTAHC, Value in Health) but may also want to consider whether a model that is not open source, with little detail on methods, is of publication standard. Clearly some models cannot be made open as they contain commercially sensitive data; however this is unlikely the case for many applications, including lung cancer screening. Open source models allow adaptation and updates for specific questions and settings, building on the previous work. Having more models available for use by researchers would also provide opportunity for more comparison between model results, as well as foster collaboration. Initiatives such as CISNET are helpful in providing such a resource, and other funders, and policy makers, may see the benefit of such an approach. Beyond CISNET, there are other collaborations highlighting the importance of a comparative approach to modeling. This includes the Mount Hood Diabetes Challenge Network, which has been comparing the methods and results of different diabetes models since 2009 (https://www.mthooddiabeteschallenge.com/). At the last Mount Hood conference, in June 2025, seven different models took part in the challenges. A different approach is gaining recognition by national decision makers, including the National Institute for Health and Care Excellence in the UK (52) and the National Medicine’s Policy in Australia (53): the use of disease-specific reference models (54). As these organizations may be tasked with evaluating numerous interventions (e.g., treatments, procedures, diagnostics, and devices) within the same disease, the use of a single model has the potential to improve efficiency in decision making, reduce methodological inconsistencies, and improve transparency. There are of course challenges with this approach, such as ensuring the capture of all important aspects of the disease and multiple decision problems as well as providing the time and resources to develop and update such models.

For LDCT screening for lung cancer, policy makers in Asia (particularly in China) are now deluged by published economic evaluations of dubious quality, and there is no doubt pressure (due to high rates of smoking and lung cancer mortality) to make extremely consequential policy decisions on the basis of this evidence. Researchers worldwide who hold intellectual property rights over sophisticated economic models for lung cancer screening should consider the potential value to society from making those models more accessible.

## Data Availability

All data are publicly available from the individual studies.

## References

[1] Duer E, Yang H, Robinson S, et al. Do we know enough about the effect of low-dose computed tomography screening for lung cancer on mortality to act? An updated systematic review, meta-analysis and network meta-analysis of randomised controlled trials 2017 to 2021. Diagn Progn Res. 2023;7(1):26. 10.1186/s41512-023-00162-0 38072977 PMC10712083

[2] Snowsill T, Yang H, Griffin E, et al. Low-dose computed tomography for lung cancer screening in high-risk populations: a systematic review and economic evaluation. Health Technol Assess. 2018;22(69):1–276. 10.3310/hta22690 PMC6304730 30518460

[3] Peters JL, Snowsill TM, Griffin E, Robinson S, Hyde CJ. Variation in model-based economic evaluations of low-dose computed tomography screening for lung cancer: a methodological review. Value Health. 2022;25:656–665. 10.1016/j.jval.2021.11.1352 35365310

[4] Marshall D, Simpson KN, Earle CC, Chu C. Potential cost-effectiveness of one-time screening for lung cancer (LC) in a high risk cohort. Lung Cancer Jun. 2001;32(3):227–236. 10.1016/s0169-5002(00)00239-7 11390004

[5] Marshall D, Simpson KN, Earle CC, Chu CW. Economic decision analysis model of screening for lung cancer. Eur J Cancer. 2001;37(14):1759–1767. 10.1016/s0959-8049(01)00205-2 11549429

[6] Kim J, Cho B, Kim S-H, Choi C-M, Kim Y, Jo M-W. Cost utility analysis of a pilot study for the Korean lung cancer screening project. Cancer Res Treat. 2022;54(3):728–736. 10.4143/crt.2021.480 34583458 PMC9296945

[7] Sheu C-C, Wang C-C, Hsu J-S, Chung W-S, Hsu H-Y, Shi H-Y. Cost-effectiveness of low-dose computed tomography screenings for lung cancer in high-risk populations: a Markov model. World J Oncol. 2024;15(4):550–561. 10.14740/wjon1882 38993243 PMC11236381

[8] Zhang T, Chen X, Li C, et al. Cost-effectiveness analysis of risk factor-based lung cancer screening program by low-dose computer tomography in current smokers in China. Cancer. 2023;15(18):4445. 10.3390/cancers15184445 PMC10527380 37760416

[9] Zhao Z, Gu S, Yang Y, et al. A cost-effectiveness analysis of lung cancer screening with low-dose computed tomography and a polygenic risk score. BMC Cancer. 2024;24(1):73. 10.1186/s12885-023-11800-7 38218803 PMC10787978

[10] Nagy B, Szilberhorn L, Gyorbiro DM, et al. Shall we screen lung cancer with low-dose computed tomography? Cost-effectiveness in Hungary. Value in health regional issues. 2023;34:55–64. 36502786 10.1016/j.vhri.2022.10.002

[11] Al Khayat M, Eijsink JFH, Postma MJ, van de Garde EMW, van Hulst M. Cost-effectiveness of screening smokers and ex-smokers for lung cancer in the Netherlands in different age groups. Eur J Health Econ. 2022;23(7):1221–1227. 34985584 10.1007/s10198-021-01422-w PMC9395469

[12] ten Berge H, Willems B, Pan X, et al. Cost-effectiveness analysis of a lung cancer screening program in the Netherlands: a simulation based on NELSON and NLST study outcomes. J Med Econ. 2024;27(1):1197–1211. 39291295 10.1080/13696998.2024.2404359

[13] ten Berge H, Ramaker D, Piazza G, et al. Shall we screen lung cancer with volume computed tomography in Austria? A cost-effectiveness modelling study. Cancer. 2024;16(15):10.3390/cancers16152623 PMC11310943 39123350

[14] Lung Cancer Policy Network. Interactive map of lung cancer screening. 2024. Available from: https://www.lungcancerpolicynetwork.com/interactive-map-of-lung-cancer-screening/ (accessed 10 December).

[15] Whynes DK. Could CT screening for lung cancer ever be cost effective in the United Kingdom? Cost Eff Resour Alloc. 2008;6:5. 10.1186/1478-7547-6-5 18302756 PMC2292150

[16] Field JK, Duffy SW, Baldwin DR, et al. The UK lung cancer screening trial: a pilot randomised controlled trial of low-dose computed tomography screening for the early detection of lung cancer. Health Technol Assess. 2016;20(40):1–146. 10.3310/hta20400 PMC4904185 27224642

[17] Hinde S, Crilly T, Balata H, et al. The cost-effectiveness of the Manchester ‘lung health checks’, a community-based lung cancer low-dose CT screening pilot. Lung Cancer. 2018;126:119–124. 10.1016/j.lungcan.2018.10.029 30527175

[18] Pyenson BS, Sander MS, Jiang Y, Kahn H, Mulshine JL. An actuarial analysis shows that offering lung cancer screening as an insurance benefit would save lives at relatively low cost. Health Aff (Millwood). 2012;31(4):770–779. 22492894 10.1377/hlthaff.2011.0814

[19] Pyenson BS, Henschke CI, Yankelevitz DF, Yip R, Dec E. Offering lung cancer screening to high-risk medicare beneficiaries saves lives and is cost-effective: an actuarial analysis. Am Health Drug Benefits. 2014;7(5):272–282. 25237423 PMC4163779

[20] Villanti AC, Jiang Y, Abrams DB, Pyenson BS. A cost-utility analysis of lung cancer screening and the additional benefits of incorporating smoking cessation interventions. PLoS One. 2013;8(8):e71379 23940744 10.1371/journal.pone.0071379 PMC3737088

[21] National Cancer Institute Cancer Intervention and Surveillance Modeling Network (CISNET). Lung cancer outcomes simulator. Available from: https://resources.cisnet.cancer.gov/registry/packages/lcos-stanford/ (accessed 28 September 2020).

[22] Criss SD, Cao P, Bastani M, et al. Cost-effectiveness analysis of lung cancer screening in the United States: a comparative Modeling study. Ann Intern Med. 2019;171(11):796–804. 31683314 10.7326/M19-0322

[23] Toumazis I, Tsai EB, Ayca Erdogan S, et al. Cost-effectiveness analysis of lung cancer screening accounting for the effect of indeterminate findings. JNCI Cancer Spectrum. 2019;3(3):pkz035 31942534 10.1093/jncics/pkz035 PMC6947892

[24] National Cancer Institute Cancer Intervention and Surveillance Modeling Network (CISNET). Lung cancer policy model. Available from: https://resources.cisnet.cancer.gov/registry/packages/lcpm-mgh/ (accessed 28 September 2020).

[25] McMahon PM, Kong CY, Bouzan C, et al. Cost-effectiveness of computed tomography screening for lung cancer in the United States. J Thorac Oncol. 2011;6(11):1841–1848. 21892105 10.1097/JTO.0b013e31822e59b3 PMC3202298

[26] National Cancer Institute Cancer Intervention and Surveillance Modeling Network (CISNET). MISCAN-lung. 2020. Available from: https://resources.cisnet.cancer.gov/registry/packages/miscan-lung-erasmus/ (accessed 28 September 2020).

[27] ten Haaf K, Tammemägi MC, Bondy SJ, et al. Performance and cost-effectiveness of computed tomography lung cancer screening scenarios in a population-based setting: a microsimulation Modeling analysis in Ontario, Canada. PLoS Med. 2017;14(2):e1002225 28170394 10.1371/journal.pmed.1002225 PMC5295664

[28] Tomonaga Y, ten Haaf K, Frauenfelder T, et al. Cost-effectiveness of low-dose CT screening for lung cancer in a European country with high prevalence of smoking-a modelling study. Lung Cancer. 2018;121:61–69. 29858029 10.1016/j.lungcan.2018.05.008

[29] Tomonaga Y, de Nijs K, Bucher HC, de Koning H, Ten Haaf K. Cost-effectiveness of risk-based low-dose computed tomography screening for lung cancer in Switzerland. Int J Cancer. 2024;154(4):636–647. 10.1002/ijc.34746 37792671

[30] National Cancer Institute Cancer Intervention and Surveillance Modeling Network (CISNET). University of michigan lung cancer screening model. Available from: https://resources.cisnet.cancer.gov/registry/packages/um-lcsc-michigan/ (accessed 28 September 2020).

[31] Pan X, Dvortsin E, Baldwin DR, et al. Cost-effectiveness of volume computed tomography in lung cancer screening: a cohort simulation based on Nelson study outcomes. J Med Econ. 2023;27(1):27–38. 38050691 10.1080/13696998.2023.2288739

[32] ten Berge H, Togka K, Pan X, et al. Cost-effectiveness of lung cancer screening with volume computed tomography in Portugal. Journal of comparative effectiveness research. 2024;e240102 39329332 10.57264/cer-2024-0102 PMC11542083

[33] Yuan J, Sun Y, Wang K, et al. Cost effectiveness of lung cancer screening with low-dose CT in heavy smokers in China. Cancer Prev Res (Phila). 2022;15(1):37–44. 34580085 10.1158/1940-6207.CAPR-21-0155

[34] Yuan J, Sun Y, Xu F, et al. Cost-effectiveness of lung cancer screening combined with nurse-led smoking cessation intervention: a population-based microsimulation study. Int J Nurs Stud. 2022;134:104319 35926265 10.1016/j.ijnurstu.2022.104319

[35] Zhao Z, Du L, Li Y, et al. Cost-effectiveness of lung cancer screening using low-dose computed tomography based on start age and interval in China: Modeling study. JMIR Public Health Surveill. 2022;8(7):e36425 35793127 10.2196/36425 PMC9301557

[36] Adair O, Lamrock F, O’Mahony JF, Lawler M, McFerran E. A comparison of international Modeling methods for evaluating health economics of colorectal cancer screening: a systematic review. Value Health. 2025;28(5):790–799. 10.1016/j.jval.2025.01.007 39880192

[37] Ten Haaf K, Bastani M, Cao P, et al. A comparative Modeling analysis of risk-based lung cancer screening strategies. J Natl Cancer Inst. 2020;112(5):466–479. 10.1093/jnci/djz164 31566216 PMC7225672

[38] Behar Harpaz S, Weber MF, Wade S, et al. Updated cost-effectiveness analysis of lung cancer screening for Australia, capturing differences in the health economic impact of NELSON and NLST outcomes. Br J Cancer. 2023;128(1):91–101. 10.1038/s41416-022-02026-8 36323879 PMC9814515

[39] Kumar V, Cohen JT, van Klaveren D, et al. Risk-targeted lung cancer screening: a cost-effectiveness analysis. Ann Intern Med. 2018;168(3):161–169. 10.7326/m17-1401 29297005 PMC6533918

[40] Black WC, Gareen IF, Soneji SS, et al. Cost-effectiveness of CT screening in the National lung screening trial. N Engl J Med 2014;371(19):1793–802. 10.1056/NEJMoa1312547 25372087 PMC4335305

[41] Goffin JR, Flanagan WM, Miller AB, et al. Cost-effectiveness of lung cancer screening in Canada. JAMA Oncol. 2015;1(6):807–813. 10.1001/jamaoncol.2015.2472 26226181

[42] Goffin JR, Flanagan WM, Miller AB, et al. Biennial lung cancer screening in Canada with smoking cessation-outcomes and cost-effectiveness. Lung Cancer. 2016;101:98–103. 10.1016/j.lungcan.2016.09.013 27794416

[43] Fabbro M, Hahn K, Novaes O, O’Gralaigh M, O’Mahony JF. Cost-effectiveness analyses of lung cancer screening using low-dose computed tomography: a systematic review assessing strategy comparison and risk stratification. PharmacoEconomics - Open. 2022;6(6):773–786. 10.1007/s41669-022-00346-2 36040557 PMC9596656

[44] Husereau D, Drummond M, Augustovski F, et al. Correction to: consolidated health economic evaluation reporting standards 2022 (CHEERS 2022) statement: updated reporting guidance for health economic evaluations. Appl Health Econ Health Policy. 2022;20(5):781–782. 10.1007/s40258-022-00743-y 35840812 PMC9385799

[45] Husereau D, Drummond M, Petrou S, et al. Consolidated health economic evaluation reporting standards (CHEERS) statement. BJOG. 2013;120(6):765–770. 10.1111/1471-0528.12241 23565948

[46] Du Y, Sidorenkov G, Heuvelmans MA, et al. Cost-effectiveness of lung cancer screening with low-dose computed tomography in heavy smokers: a microsimulation modelling study. Eur J Cancer. 2020;135:121–129. 10.1016/j.ejca.2020.05.004 32563896

[47] Gómez-Carballo N, Fernández-Soberón S, Rejas-Gutiérrez J. Cost-effectiveness analysis of a lung cancer screening programme in Spain. Eur J Cancer Prev. 2022;31(3):235–244. 10.1097/cej.0000000000000700 34406177

[48] Husereau D, Drummond M, Augustovski F, et al. Consolidated health economic evaluation reporting standards (CHEERS) 2022 explanation and elaboration: a report of the ISPOR CHEERS II good practices task force. Value Health. 2022;25(1):10–31. 35031088 10.1016/j.jval.2021.10.008

[49] Brennan A, Chick SE, Davies R. A taxonomy of model structures for economic evaluation of health technologies. Health Econ. 2006;15(12):1295–1310. 16941543 10.1002/hec.1148

[50] Stahl JE. Modelling methods for pharmacoeconomics and health technology assessment: an overview and guide. PharmacoEconomics. 2008;26(2):131–148. 18198933 10.2165/00019053-200826020-00004

[51] Dahabreh IJ, Trikalinos TA, Balk EM, Wong JB. Recommendations for the conduct and reporting of Modeling and simulation studies in health technology assessment. Ann Intern Med. 2016;165(8):575–581. 27750326 10.7326/M16-0161

[52] National Institute for Health and Care Excellence (NICE). Use of disease-specific reference models in economic evaluations: NICE position statement (ECD18). 2025. https://www.nice.org.uk/corporate/ecd18

[53] Health Technology Assessment Policy and Methods Review Reference Committee. Accelerating access to the best medicines for Australians now into the future: A review of Australia’s health technology assessment policies and methods for the Australian Government. 2024. https://www.health.gov.au/sites/default/files/2024-09/health-technology-assessment-policy-and-methods-review-final-report_0.pdf

[54] Haji Ali Afzali H, Karnon J. Expediting patient access to new health technologies: role of disease-specific reference models. Value Health. 2021;24(6):755–758. 34119072 10.1016/j.jval.2020.12.013

